# A Large, Severely Obstructive, Calcified Mass in the Midsegment of Aortic Arch

**DOI:** 10.1055/s-0039-3401020

**Published:** 2020-02-04

**Authors:** Brijesh Patel, Mahek Shah, Apurva Vyas, Timothy Misselbeck, James A. Burke

**Affiliations:** 1 Department of Cardiology, Lehigh Valley Hospital Network, Allentown, Pennsylvania; 2 West Virginia University, Morgantown, West Virginia; 3 Department of Cardiothoracic Surgery, Lehigh Valley Hospital Network, Allentown, Pennsylvania

**Keywords:** aortic arch, calcified lesion, angiography

## Abstract

Severe obstructive lesions in the aortic arch are rare. Crossing such lesions poses additional challenges in patients who require cardiac catheterizations. Oftentimes, specialized catheters are required to negotiate the lesion. Herein, we are reporting a series of case images that illustrate a severe lesion in the aortic arch during coronary angiography.

A 69-year-old woman was referred for cardiac catheterization owing to non-ST elevation myocardial infarction. Two weeks prior to this admission, she was treated for pontine stroke and had mild right-sided residual symptoms. She had been experiencing abdominal pain after meals, claudication with moderate effort. On physical examination, she had faint lower extremity and left radial pulses, and normal right radial arterial pulses. The left cardiac catheterization was accessed using a 6-French (F) sheath via the right femoral artery. Then, a 0.035 in J-wire met the resistance from a large chunk of calcified lesion at the level of midaortic arch ( Fig. 1 ). Instead, a hydrophilic guidewire, ZipWire (Boston Scientific, Marlborough, MA) was used to cross the lesion over which a QuickCross catheter (Spectranetics, Colorado Springs, CO) was guided. Then, a 0.035 in VersaCore (Abbott, Abbott Park, IL) workhorse wire was exchanged via the catheter. Coronary angiography was performed with 4-F Judkins' right catheter. It revealed calcified coronary vessels but no significant intraluminal stenosis ( Figs. 2 and 3 ). Central aortic pressures before and after the lesion were 155/51 and 79/47, respectively. Two days later, she underwent dedicated computer tomography for the aorta. It showed an isolated, large calcified lesion with near total occlusion. Mild, diffuse atherosclerosis was noted in the ascending and descending aorta ( Figs. 4 5 6 ).

**Fig. 1:**
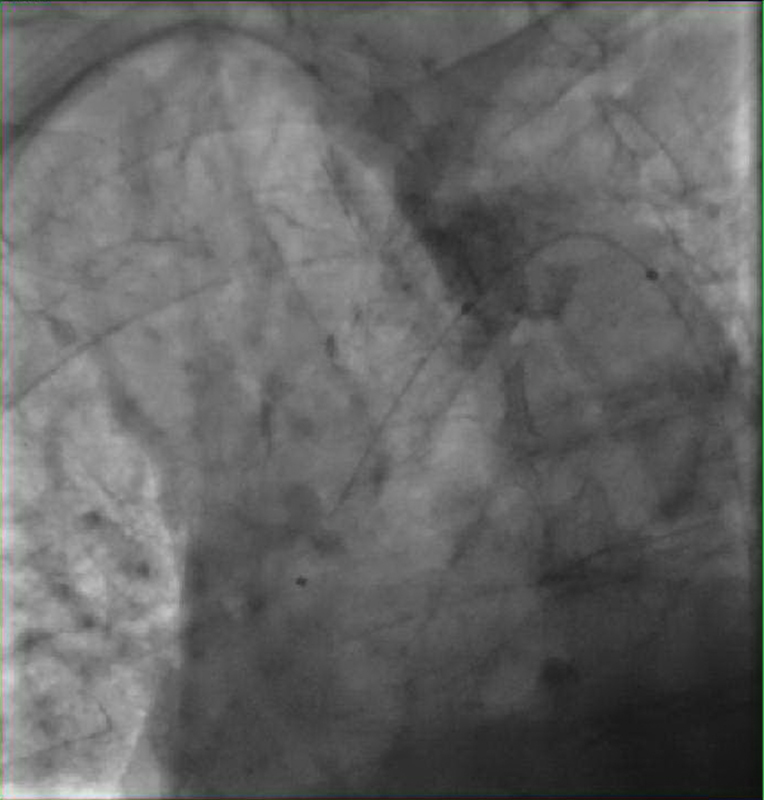
The calcified lesion was crossed with Zipwire, and QuickCross wire was passed over it.

**Fig. 2:**
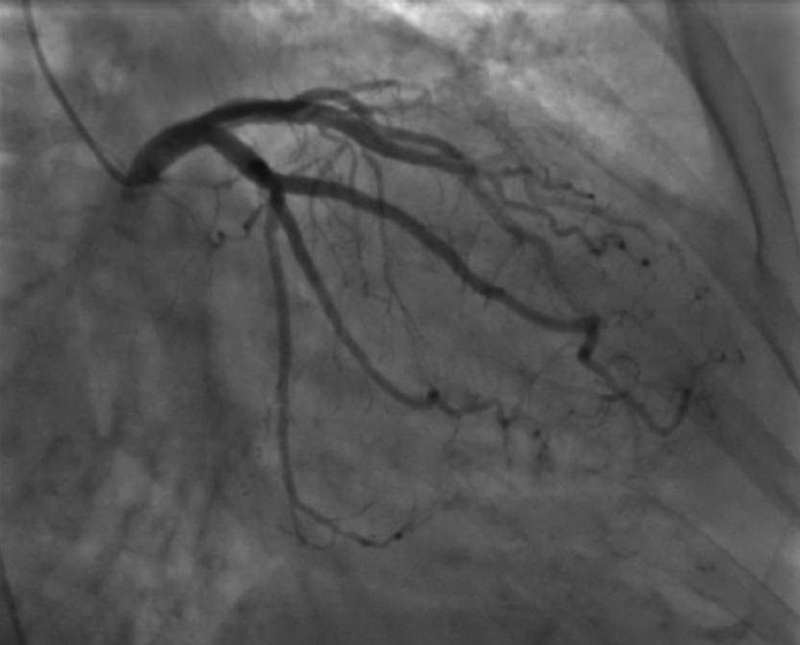
The left coronary artery system shows minimal intraluminal coronary artery disease.

**Fig. 3:**
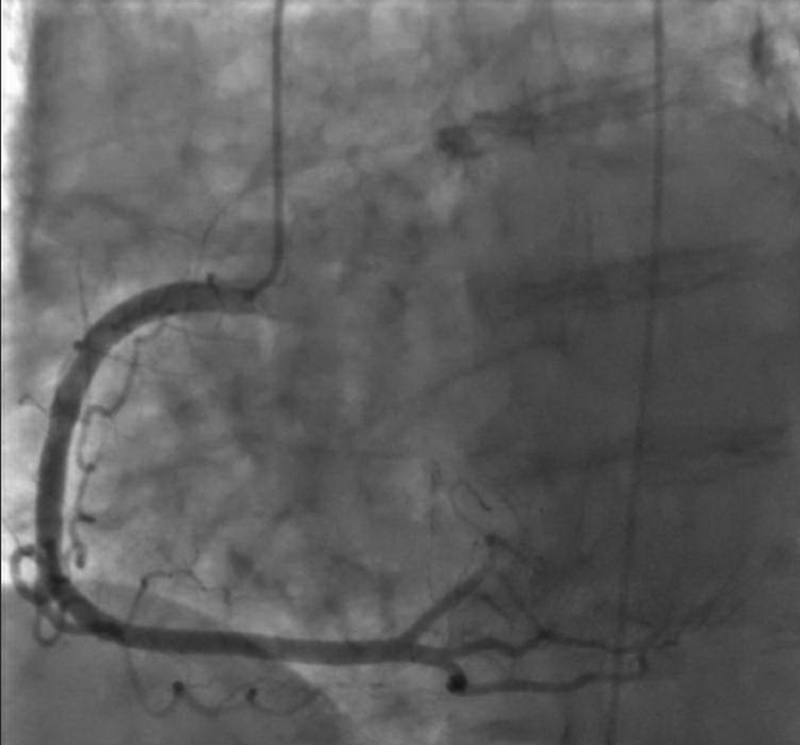
The right coronary artery has no intraluminal coronary artery disease.

**Fig. 4:**
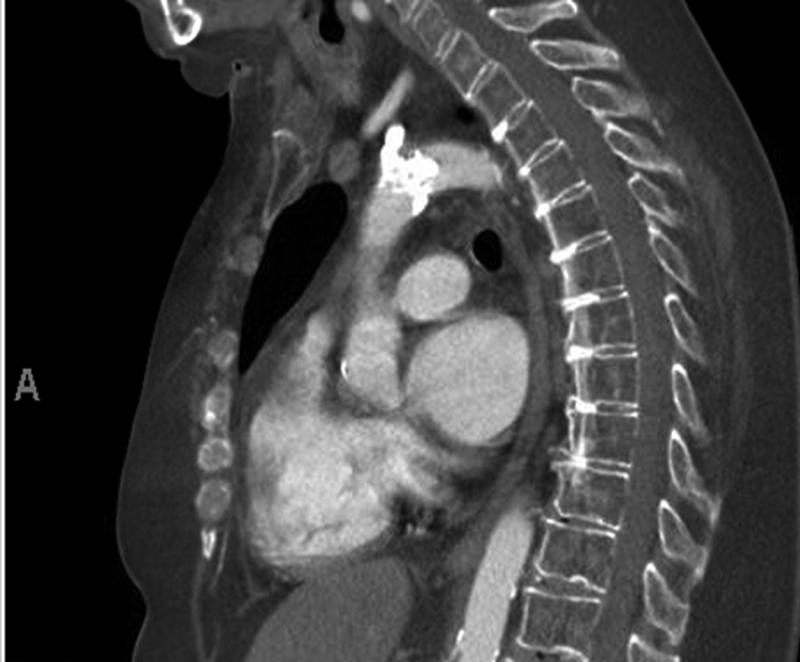
A sagittal view of the aorta showing a calcified lesion in the midaortic arch.

**Fig. 5:**
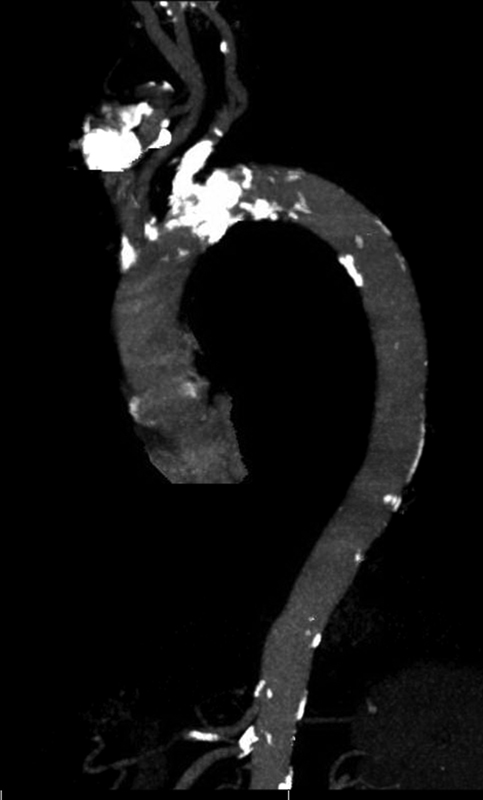
A candy cane view of the aorta showing calcified lesion in the midsegment of aortic arch and branch vessels.

**Fig. 6:**
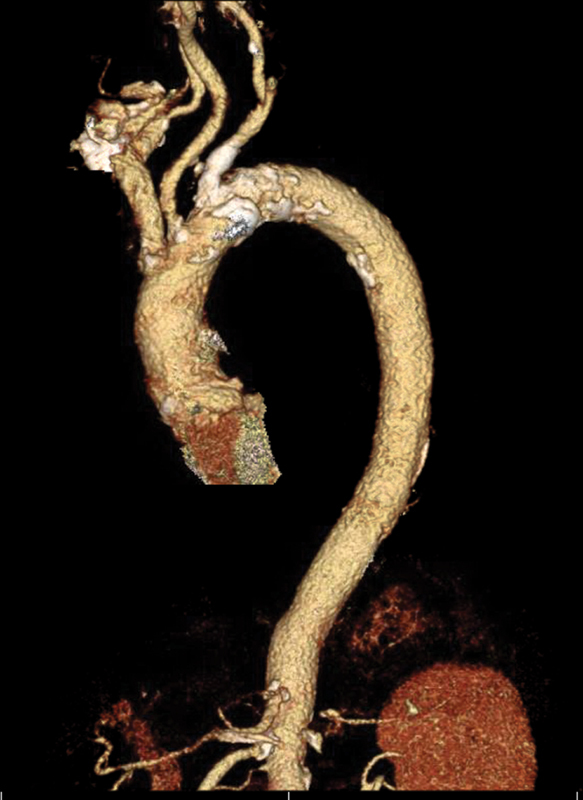
A 3D reconstruction of the candy cane view of the aorta showing the calcified lesion in the midsegment of aortic arch and branch vessels.

## Notes

Editor's Questions

1. *Did you suspect an obstructive lesion because of the pulse differences on initial physical exam?*
  We did not suspect an obstructive lesion to this extent before the procedure. It was certainly in differential diagnosis.
2. *Was there an audible bruit in the chest?*
  There was no audible bruit in the chest.
3. *Was there abundant calcification on the initial chest X-ray (CXR) that might have made you suspect what you later found?*
  On cardiac catheterization, there was obvious calcification, but we did not expect it to very obstructive. Among all radiologic imaging, computed tomography (CT) angiography correlated the best but this was postcardiac catheterization.
4. *Why did you not catheterize via the right radial, avoiding that severe obstructive arch lesion?*
  As mentioned early, we did not expect such a finding on pre-cath assessment. We thought that the lesion/gradient would be better assessed if we access through femoral artery. In retrospect, the right radial artery access would have been optimal coronary angiography but no technically feasible to assess the lesion or gradient.
5. *Were you not concerned about the danger of embolization?*
  Yes, we performed the procedure cautiously, though we acknowledged that there is a higher risk of embolization with type of lesion.
6. *Did you treat the obstructive lesion in any way? You have a gradient of nearly 80 mm Hg. This is like a very severe coarctation. One can expect blood pressure (BP) and renal consequences, in addition to the patient's claudication. Please explain* .
  The lesion was not amenable to percutaneous interventions. Therefore, we consulted cardiothoracic surgery service for further recommendation. However, the patient did not want to pursue any surgical recommendations.

